# Arylesterase Activity of HDL Associated Paraoxonase as a Potential Prognostic Marker in Patients With Sepsis and Septic Shock—A Prospective Pilot Study

**DOI:** 10.3389/fmed.2020.579677

**Published:** 2020-10-22

**Authors:** Alexander C. Reisinger, Max Schuller, Michael Holzer, Julia T. Stadler, Gerald Hackl, Florian Posch, Gunther Marsche, Harald Sourij, Robert Ekart, Kathrin Eller, Philipp Eller

**Affiliations:** ^1^Intensive Care Unit, Department of Internal Medicine, Medical University of Graz, Graz, Austria; ^2^Division of Nephrology, Department of Internal Medicine, Medical University of Graz, Graz, Austria; ^3^Division of Pharmacology, Otto Loewi Research Center for Vascular Biology, Immunology and Inflammation, Medical University of Graz, Graz, Austria; ^4^Division of Oncology, Department of Internal Medicine, Medical University of Graz, Graz, Austria; ^5^Division of Endocrinology and Diabetology, Department of Internal Medicine, Medical University of Graz, Graz, Austria; ^6^Department of Dialysis, Clinic for Internal Medicine, University Clinical Centre Maribor, Maribor, Slovenia

**Keywords:** sepsis, ICU, arylesterase activity, paraoxonase (PON), paraoxonase (PON1), dyslipideamia, high density lipoprotein (HDL)

## Abstract

**Background:** High-density lipoprotein (HDL) plays an essential role in the immune system and shows effective antioxidative properties. We investigated correlations of lipid parameters with the sequential organ failure assessment (SOFA) score and the prognostic association with mortality in sepsis patients admitted to intensive care unit (ICU).

**Methods:** We prospectively recruited consecutive adult patients with sepsis and septic shock, according to sepsis-3 criteria as well as non-sepsis ICU controls.

**Results:** Fifty-three patients with sepsis (49% with septic shock) and 25 ICU controls without sepsis were enrolled. Dyslipidemia (HDL-C < 40 mg/l) was more common in sepsis compared to non-sepsis patients (85 vs. 52%, *p* = 0.002). Septic patients compared to controls had reduced HDL-C (14 vs. 39 mg/l, *p* < 0.0001), lower arylesterase activity of the antioxidative paraoxonase of HDL (AEA) (67 vs. 111 mM/min/ml serum, *p* < 0.0001), and a non-significant trend toward reduced cholesterol efflux capacity (9 vs. 10%, *p* = 0.091). We observed a strong association between higher AEA and lower risk of 28-day [per 10 mM/min/ml serum increase in AEA: odds ratio (OR) = 0.76; 95% CI, 0.61–0.94; *p* = 0.01) and ICU mortality (per 10 mM/min/ml serum increase in AEA: OR = 0.71, 95% CI, 0.56–0.90, *p* = 0.004) in the sepsis cohort in univariable logistic regression analysis. AEA was confirmed as an independent predictor of 28-day and ICU mortality in multivariable analyses. AEA discriminated well-regarding 28-day/ICU mortality in area under the receiver operating characteristic curve (AUROC) analyses. In survival analysis, 28-day mortality estimates were 40 and 69% with AEA ≥/< the 25th percentile of AEA's distribution, respectively (log-rank *p* = 0.0035).

**Conclusions:** Both compositional and functional HDL parameters are profoundly altered during sepsis. In particular, the functionality parameter AEA shows promising prognostic potential in sepsis patients.

## Introduction

Sepsis is a global medical threat and one of the leading causes of death worldwide (1, 2). The current “sepsis-3” definition characterizes sepsis as “a life-threatening organ dysfunction caused by a dysregulated host response to infection” (1). Clinical diagnosis and presence of new organ dysfunction currently remain the gold standard in diagnosing sepsis (3). Unfortunately, clinical diagnosis is unreliable in diagnosing septic patients admitted to the ICU. One *post hoc* analysis study revealed that 13% of patients who were considered to have sepsis by the treating clinician had no infection at all (4). Because of these limitations, easy to measure and broadly available biomarkers are necessary that may aid doctors in diagnosing and predicting outcome of septic patients. High-density lipoprotein (HDL) particles are primarily known for their role in cardiovascular disease and reverse cholesterol transport (5, 6). However, HDL also transport cholesterol to adrenal glands for steroid synthesis and play an important role in the innate and adaptive immune system. HDL can bind lipopolysaccharides (LPS) and lipoteichoic acid (LTA) and transport these lipids to the liver for uptake and neutralization (7–9). HDL also have antioxidative properties, can activate the endothelial nitric oxide synthase (eNOS), and reduce adhesion molecule expression in vascular endothelial cells (6, 8, 10–12). Antioxidative mechanisms are altered in dysfunctional HDL, which has been identified in several chronic diseases including end stage renal disease, psoriasis, rheumatoid arthritis, and diabetes (13–15). Qualitative changes of HDL such as cholesterol efflux capacity (CEC) and the antioxidative and anti-inflammatory arylesterase activity of HDL-associated paraoxonase (AEA) have, to date, sparsely been investigated in septic patients (16–18). To determine whether functionality parameters associated with HDL predict sepsis severity and outcome, we performed a prospective study investigating the quantitative and qualitative changes of HDL of critically ill patients admitted to the medical ICU.

## Materials and Methods

### Study Population and Study Design

#### Sepsis and Control Cohort

In this prospective study, we recruited adult (i.e., ≥18 years) patients with sepsis and septic shock who presented at the ICU of the Department of Internal Medicine at the Medical University of Graz, Austria between 2018 and 2019. Sepsis was defined according to sepsis-3 criteria, which included a suspected infection by the treating critical care physician and an increase in the sequential organ failure assessment (SOFA) score by at least 2 points (1). The baseline SOFA score was deduced from previous medical records. Septic shock was defined as vasopressor therapy to maintain a mean arterial pressure (MAP) ≥65 mmHg in the absence of hypovolemia and/or after adequate fluid resuscitation and a lactate level of >2 mmol/L. Patients were excluded if they were (a) older than 100 years, (b) in palliative or comfort terminal care, (c) pregnant, or (d) diagnosed with acquired immunodeficiency syndrome (AIDS). Moreover, we included a control cohort of consecutive adult patients who were admitted to the ICU but did not have sepsis or bacteremia at the time of sample acquisition. The study protocol was approved by the Institutional Review Board (IRB) of the Medical University of Graz, Austria (30-258 ex 17/18). The study is registered in the German Clinical Trials Register (DRKS-ID #DRKS00015315) and complied with the Declaration of Helsinki. Written informed consent was obtained from all conscious participants. In comatose non-survivors, the institutional review board (IRB) waived the need for written informed consent.

### Laboratory Analyses

Blood cell count, serum creatinine, bilirubin, C-reactive protein (CRP), procalcitonin (PCT), albumin, serum levels of total cholesterol, HDL cholesterol (HDL-C), triglyceride (TG), apolipoprotein A-I (ApoA1), and apolipoprotein B (ApoB) levels were measured using a Sysmex (Sysmex Austria GmbH), Cobas (Roche Diagnostics), or BN II analyzer (Siemens Healthcare Diagnostics) as appropriate. Low-density lipoprotein (LDL) cholesterol was calculated according to the Friedewald formula. ApoB-depleted serum was prepared by the addition of 40 μl of polyethylene glycol (20% in 200 mmol/L glycine buffer) to 100 μl of serum. Samples were incubated for 20 min, and the HDL-containing supernatants (ApoB-depleted serum) were recovered after centrifugation (9,703 g, 20 min, 4°C) and stored at −80°C (6). Qualitative parameters of HDL metabolism such as cholesterol efflux capacity (CEC) and arylesterase activity of HDL-associated paraoxonase (AEA) were contemporaneously measured from ApoB-depleted sera from all patients after finishing recruitment. Ca^2+^-dependent AEA was determined by a photometric assay using phenylacetate as substrate as described (6). Briefly, ApoB-depleted serum was added to 200 μl of buffer containing 100 mmol/L Tris, 2 mmol/L CaCl_2_ (pH 8.0), and 1 mmol/l phenylacetate. The rate of hydrolysis of phenylacetate was monitored at 270 nm. The enzymatic activity was calculated with the Beer–Lambert law from the molar extinction coefficient of 1,310 L mol^−1^ cm^−1^. CEC was quantified with a previously established methodology (6, 19). To correct for any possible interassay variation across plates, we included a serum control on each plate and normalized values for serum samples from patients to this value. Briefly, J774 cells, a mouse macrophage cell line, were plated and labeled for 24 h with 1 μCi/ml [3H]cholesterol (Perkin Elmer, Boston, MA). J774 cells express very low levels of ATP-binding cassette transporter A1 (ABCA1), an important pathway of cholesterol efflux from macrophages. To upregulate ABCA1, cells were stimulated for 6 h with serum-free Dulbecco's modified Eagle's medium (DMEM) containing 0.3 mmol/L 8-(4-chlorophenylthio)-cyclic AMP (Sigma, Darmstadt, Germany). Upon cAMP treatment, ABCA1-dependent cholesterol efflux increases threefold to about 40%, while passive diffusion accounts for 50% and scavenger receptor B1 (SR-BI) for 10% (20). After labeling, cells were washed, and [3H]cholesterol efflux was determined by incubating cells for 4 h with 2.8% ApoB-depleted serum as described (19). Liquid scintillation counting was performed to measure the efflux of radioactive cholesterol from cells. Radioactive cholesterol quantity incorporated into cellular lipids was assessed by means of isopropanol extraction of cells not exposed to the patients' serum. All steps were performed in the presence of a concentration of 2 μg/ml of the acyl CoA cholesterol acyltransferase inhibitor Sandoz 58-035 (Sigma, Darmstadt, Germany).

### Pre-specification of Analyses

For all analyses, we considered the following lipid parameters: (1) HDL-C, (2) TG, (3) AEA, (4) CEC, and (5) ApoA1. Primary measure of interest as per the study protocol was the prevalence of dyslipidemia, defined by an HDL-C level <40 mg/l. Other quantities of interest were (1) the correlation between lipid parameters and the SOFA score, (2) the prognostic association between lipid parameters and 28-day or ICU all-cause mortality, and (3) differences between sepsis patients and controls.

### Statistical Analyses

All statistical analyses were performed with SPSS 26 (SPSS Inc, Chicago, IL, USA) and Stata 15.0 (Stata Corp., Houston, TX, USA). Continuous variables were summarized as medians (25–75th percentile) and categorical variables as absolute frequencies (%). Associations between variables were analyzed with cross-tabulations, Mann–Whitney *U*-tests, χ^2^-tests, and Fisher's exact tests, as appropriate. Correlations were computed with Spearman's rank-based correlation coefficient. In all analyses, we treated lipid function parameters as continuous variables. The prognostic associations between 28-day/ICU mortality and lipid parameters, the SOFA score, and other potential baseline predictors were quantified with univariable logistic regression. For multivariable adjustment, we always considered the SOFA score and, additionally, all variables that were univariable associated with the outcome with a *p* ≤ 0.05. For survival analysis (outcome: 28-day mortality), we used Kaplan–Meier functions estimators and log-rank tests. Significance level was defined at 0.05. Formal adjustment for multiple testing was not performed. The full analysis code is available on request from the corresponding author.

## Results

### Baseline Characteristics

Fifty-three sepsis patients, of whom 26 (49%) were in septic shock, and 25 controls without signs of sepsis or bacteremia were prospectively enrolled (Table 1). Briefly, in the sepsis cohort, the median SAPS-3 score was 65 (56–75) points, the median SOFA score was 9 (7–13) points, and the median lactate level was 2.4 mmol/L (1.2–5.1). Blood cultures were drawn in 94% of cases, and thereof, 52% were positive with 30% Gram-positive, 18% Gram-negative bacteria, 2% mixed infections, and 2% fungi. Nosocomial infections accounted for a minority of 9.4%. The most common focus was pneumonia (42%), followed by abdominal sepsis (17%), and urogenital tract infection (11%). Eighty-five percent of patients suffering from sepsis needed catecholamine treatment, and 45% required renal replacement therapy. We provided catecholamine therapy for patients on median 2 days (1–7). Median length of stay in the ICU was 6 (3–10) days, and median length of stay in the hospital was 16 (6.5–25.5) days. ICU mortality and 28-day mortality were 36 and 47%, respectively (Table 1). Non-sepsis control patients received catecholamine therapy on median 1 (0–2) day, and median ICU and hospital length of stay were 3 (2–6) and 15 (8–27) days, respectively. As expected, inflammatory markers including white blood count (WBC; 14.9 vs. 9.1 g/L, *p* = 0.011), CRP (213 vs. 12 mg/l, *p* < 0.0001; normal range <5 mg/l), and PCT (8.8 vs. 0.15 ng/ml, *p* < 0.0001) were higher in sepsis patients compared to ICU controls. Sepsis patients were slightly younger at 66 compared to 72 years of age (*p* = 0.012) but had similar rates of pre-existing diabetes or liver disease.

**Table 1:** Baseline characteristics and outcomes of the study population.

**Variable**	**Sepsis cohort (*n* = 53)**	**Control cohort (*n* = 25)**	***p*-value**	**Normal range**
**Demographics and pre-medication**				
Age (years)	66 (50–75)	72 (65–79)	0.012	N/A
Body mass index (kg/m^2^)	25.8 (23.4–29.8)	27.8 (23.9–30.2)	0.483	18.5–24.9
Female sex	21 (40%)	15 (60%)	0.144	N/A
Anti-diabetic therapy	12 (23%)	8 (32%)	0.413	N/A
Statin therapy	15 (28%)	7 (28%)	1.000	N/A
Diabetes	15 (28%)	8 (32%)	0.793	N/A
Liver disease	3 (6%)	2 (8%)	0.653	N/A
Propofol therapy before sample acquisition	3 (6%)	7 (28%)	0.010	N/A
Enteral/parenteral nutrition before sample acquisition	5 (9%)	1 (4%)	0.658	N/A
Mechanical ventilation at sample acquisition	22 (42%)	9 (36%)	0.805	N/A
Time to sample acquisition (h)	3.3 (0.7–16.5)	3.6 (0.4–11.7)	0.490	N/A
**Quantitative lipid parameters**				
HDL cholesterol (mg/l)	14 (7–33)	39 (33–55)	<0.0001	>40
Triglycerides (mg/l)	162 (105–274)	115 (80–145)	0.006	<150
Total cholesterol (mg/l)	106 (84–130)	114 (96–156)	0.193	<200
LDL cholesterol (mg/l)^a^	57 (28–74)^b^	51 (36–77)	0.793	N/A
Apolipoprotein A-I (mg/l)	60 (31–90)	103 (85–130)	<0.0001	95–200
Apolipoprotein B (mg/l)	67 (48–84)	66 (51–77)	0.991	50–150
**Qualitative lipid parameters**				
Arylesterase activity (AEA) (mM/min/ml serum)	66.5 (40.9–89.5)	111.2 (80.4–152.7)	<0.0001	158.5 ± 15.5^c^
Cholesterol efflux capacity (%)	9.2 (7.6–11.0)	9.9 (9.1–12.7)	0.091	13.2 ± 1.1^c^
**Laboratory covariables**				
White blood count (g/L)	14.9 (9.1–26.5)	9.1 (6.6–13.5)	0.011	4.4–11.3
Hemoglobin (g/dl)	10.8 (8.7–13.0)	10.8 (8.6–13.3)	0.672	13–17.5
Platelets (g/L)	164 (86–267)	180 (133–243)	0.312	140–440
C-reactive protein (mg/l)	213 (119–309)	12 (4–31)	<0.0001	<5.0
Procalcitonin (ng/ml)	8.8 (1.2–35.1)	0.15 (0.06–0.28)	<0.0001	<0.5
Serum bilirubin (mg/l)	0.9 (0.5–2.3)	0.4 (0.3–0.8)	0.002	0.1–1.2
Serum creatinine (mg/l)	2.3 (1.6–4)	1.2 (0.9–2.3)	0.003	<1.2
Serum albumin (g/dl)	2.9 (2.4–3.2)	3.7 (2.4–3.2)	<0.0001	3.5–5.3
**Illness severity and outcomes**				
SOFA score (points)	9 (7–13)	5 (3–9)	<0.0001	0
Catecholamine therapy	45 (85%)	13 (52%)	0.004	N/A
ICU length of stay (days)	6 (3–10)	3 (2–6)	0.031	N/A
Hospital length of stay (days)	16 (7–26)	15 (8–27)	0.940	N/A
28-day mortality	25 (47%)	4 (16%)	0.011	N/A
ICU mortality	19 (36%)	4 (16%)	0.110	N/A

*Data are reported as medians (25–75th percentile) or absolute frequencies (%)*.

*HDL, high-density lipoprotein; ICU, intensive care unit; LDL, low-density lipoprotein; N/A, not applicable; SOFA, sequential organ failure assessment*.

a *LDL cholesterol calculated according to the Friedewald formula*.

b *49 values*.

c *Mean ± SD, data derived from eight healthy controls (five male/three female; median age, 49)*.

### Prevalence and Patterns of Dyslipidemia

**Figure 1:**
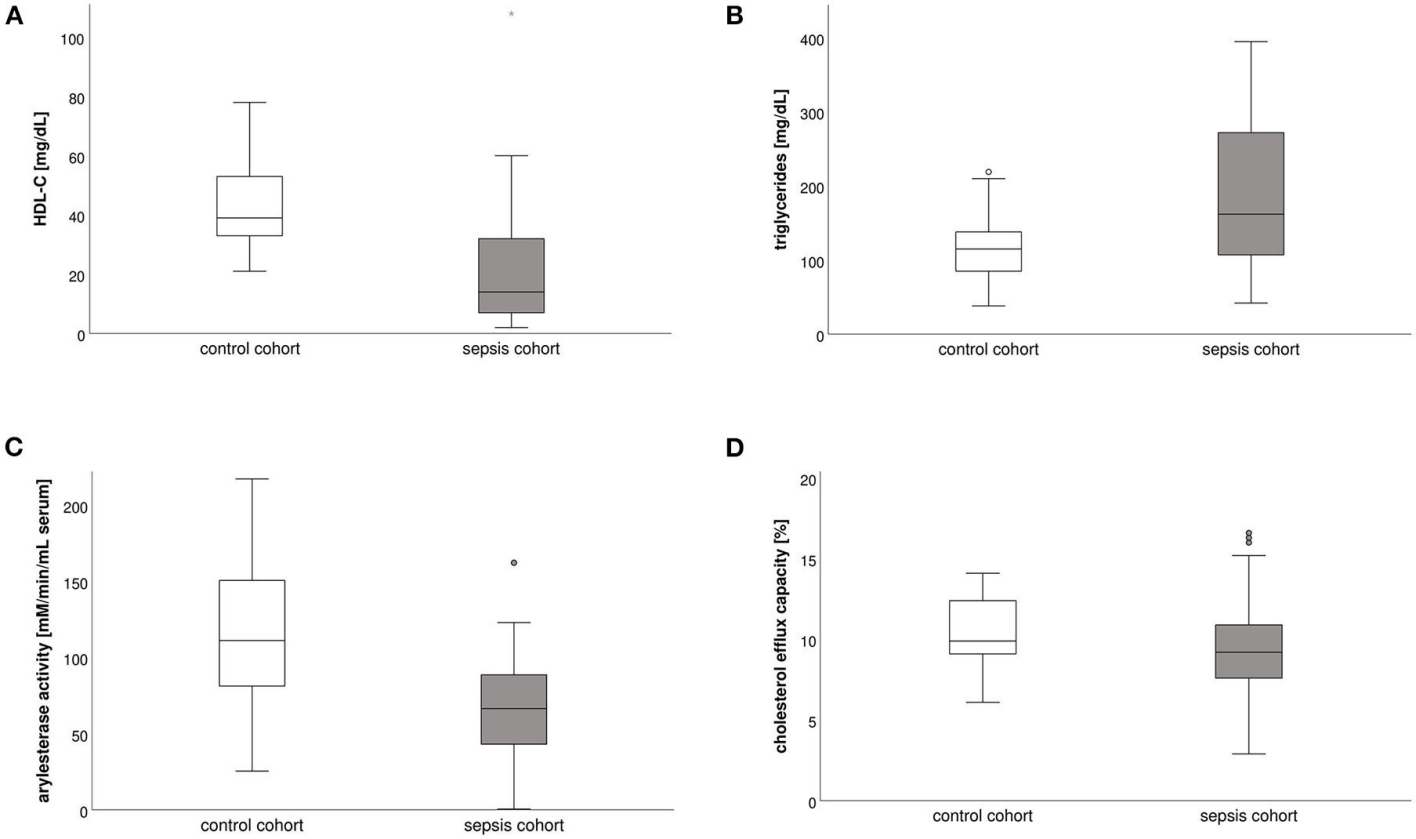
Boxplots of compositional and functional lipid parameters in the sepsis (*n* = 53; gray box plot bars) and control cohort (*n* = 25; white box plot bars). **(A)** HDL-cholesterol levels (mg/l); **(B)** triglyceride levels (mg/l); **(C)** arylesterase activity (mM/min/ml serum); **(D)** cholesterol efflux capacity (%). AEA, arylesterase activity; HDL-C, high-density lipoprotein cholesterol.

The prevalence of dyslipidemia, as defined by a serum HDL-C <40 mg/l, was 85% [95% confidence interval (CI), 72–93] in the sepsis cohort and 52% (95% CI, 31–72) in the control cohort (*p* = 0.002). Patients with sepsis compared to the control cohort had reduced HDL-C levels at 14 (7–33) compared to 39 (33–55) mg/l (*p* < 0.0001), lower AEA at 67 vs. 111 mM/min/ml serum (*p* < 0.0001), and decreased but not statistically significantly different CEC at 9% compared to 10% (*p* = 0.091; Table 1, Figure 1). In the sepsis cohort, serum triglyceride [162 mg/l (105–274)] and ApoB [67 mg/l (48–84)] levels were largely within normal range, while ApoA1 levels were altered [60 mg/l (31–90)].

### Lipid Parameters and Sepsis Severity

In the sepsis cohort, several lipid parameters were significantly correlated with sepsis severity as represented by the SOFA score. More specifically, higher SOFA scores (indicative of more severe organ dysfunction) were inversely correlated with HDL-C levels (*r* = −0.31, *p* = 0.03) and AEA (*r* = −0.32, *p* = 0.02), while no correlation was found with CEC (*r* = −0.22, *p* = 0.11) or TG levels (*r* = 0.11, *p* = 0.42), respectively. Otherwise, lower HDL-C in septic patients correlated significantly with AEA (*r* = 0.60, *p* < 0.0001), CEC (*r* = 0.54, *p* < 0.0001), serum bilirubin (*r* = −0.35, *p* = 0.01) and albumin levels (*r* = 0.59, *p* < 0.0001) but not with C-reactive protein or serum creatinine levels (Table 2). In the control cohort, the SOFA score did not correlate with HDL-C (*r* = −0.17, *p* = 0.41), AEA (*r* = −0.23, *p* = 0.28), CEC (*r* = 0.16, *p* = 0.46), or TG (*r* = 0.15, *p* = 0.48). On the other hand, HDL-C correlated with AEA (*r* = 0.49, *p* = 0.01) and CEC (*r* = 0.50, *p* = 0.01).

**Table 2:** Correlation matrix of lipid parameters and selected covariables in the sepsis cohort.

	**ApoA1**	**TG**	**AEA**	**CEC**	**SOFA**	**Bilirubin**	**CRP**	**Creatinine**	**Albumin**
HDL-C	0.803***	−0.491***	0.600***	0.537***	−0.308*	−0.345*	−0.168	−0.250	0.593***
Apolipoprotein A-I		−0.299*	0.717***	0.688***	−0.367**	−0.365**	−0.113	−0.161	0.650***
Triglycerides			−0.071	0.050	0.114	0.078	0.385**	−0.008	−0.425**
Arylesterase activity				0.716***	−0.324*	−0.185	−0.033	−0.183	0.612***
Cholesterol efflux capacity					−0.221	−0.180	0.105	−0.125	0.581***
SOFA score						0.429**	0.097	0.310*	−0.159
Bilirubin							−0.109	0.098	−0.200
C-reactive protein								0.099	−0.281*
Creatinine									−0.239

Data are Spearman's rank-based correlation coefficients (*p < 0.05, **p < 0.01, ***p < 0.001).

*AEA, arylesterase activity; ApoA1, apolipoprotein A-I; CEC, cholesterol efflux capacity; CRP, C-reactive protein; HDL, high-density lipoprotein; SOFA, sequential organ failure assessment; TG, triglycerides*.

### Lipid Parameters and Sepsis Mortality

**Figure 2:**
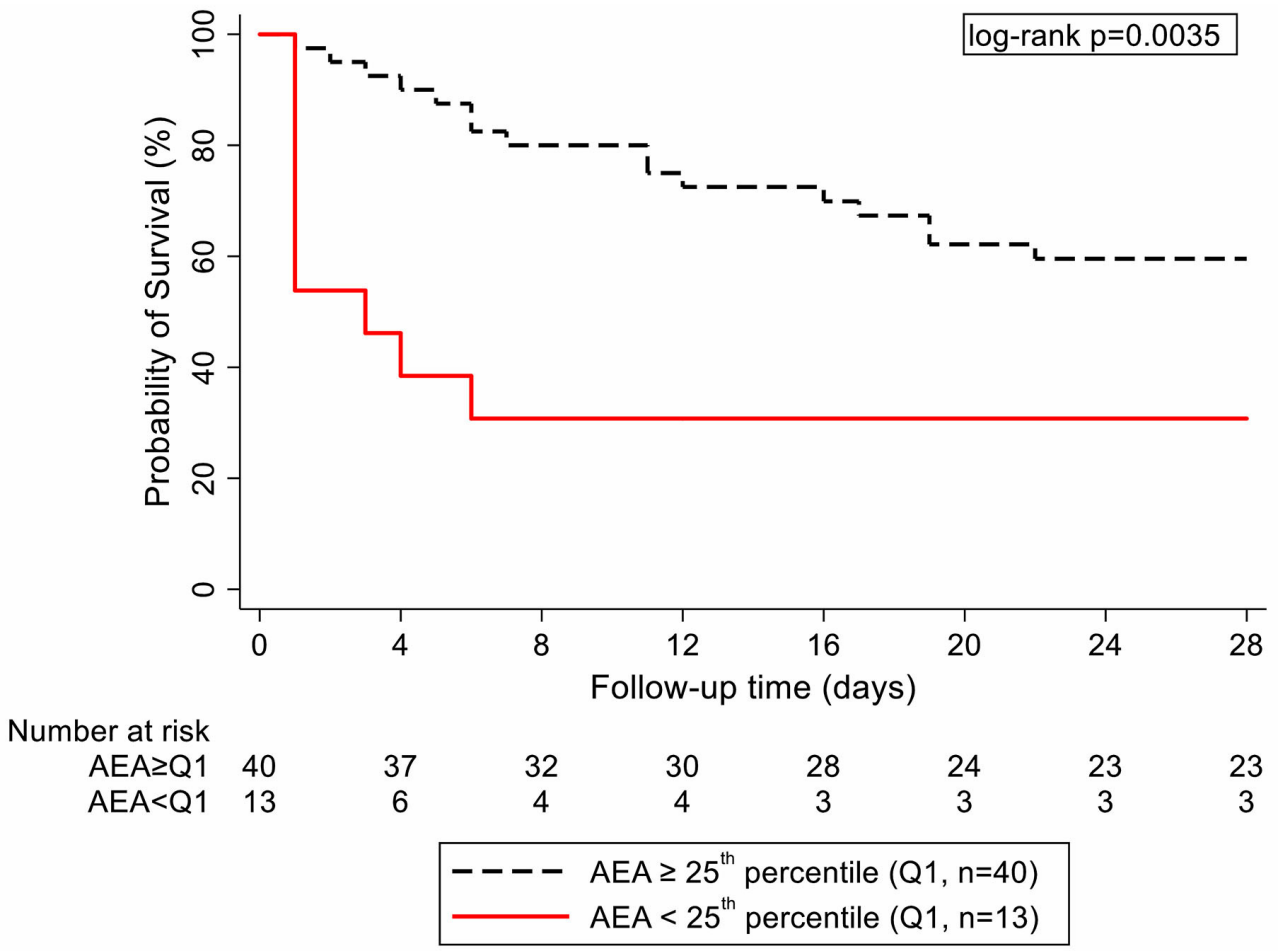
Kaplan–Meier curves of critically ill sepsis patients in dependence of arylesterase activity [empirical cutoff at the 25th percentile of its distribution: lowest quartile (red curve) vs. higher quartiles (dashed black curve)]. AEA, arylesterase activity.

The outcome of 28-day mortality occurred in 25 (47%) of the 53 patients. In univariable logistic regression, altered lipid parameters partly predicted increased sepsis mortality. In detail, while HDL-C, TG, and ApoA1 levels were not associated with 28-day mortality, a strong association between a higher AEA and a lower risk of 28-day mortality was observed [per 10 mM/min/ml serum increase: odds ratio (OR) = 0.76; 95% CI, 0.61–0.94; *p* = 0.01; Table 3]. Depending on chosen cutoffs, AEA either achieved high sensitivity and low specificity or vice versa for predicting 28-day and ICU mortality (Supplementary Tables 1, 2). On an absolute scale, 28-day mortality estimates were 40 and 69% in patients with AEA ≥ and < an empirical cutoff at the 25th percentile of AEA's distribution, respectively (log-rank *p* = 0.0035, Figure 2). Higher cholesterol levels (per 10 mg/l increase: OR = 0.90; 95% CI, 0.79–1.02; *p* = 0.09) and higher CEC (per 10% increase: OR = 0.20; 95% CI, 0.03–1.45; *p* = 0.11) were not statistically significantly associated with a lower risk of 28-day mortality with the number of patients and events we had (Table 3). Other univariable predictors of increased 28-day mortality were higher age, higher CRP levels, and lower albumin levels. For ICU mortality (*n* = 19 deaths in the ICU (36%), univariable modeling results for lipid parameters were broadly similar, with an even stronger association for AEA (per 10 mM/min/ml serum increase: OR = 0.71; 95% CI, 0.56–0.90; *p* = 0.004) and CEC (per 10% increase: OR = 0.11; 95% CI, 0.01–1.03; *p* = 0.05, Table 3). AEA was subsequently confirmed as an independent predictor of 28-day mortality and ICU mortality in multivariable analyses (Table 4). The SOFA score emerged as a poor discriminator of 28-day mortality [area under the receiver operating characteristic curve (AUROC) = 0.62; 95% CI, 0.46–0.78] but as a strong discriminator of ICU mortality (AUROC = 0.78, 95% CI, 0.65–0.91). In contrast, AEA discriminated well for both 28-day mortality (AUROC = 0.71; 95% CI, 0.57–0.85) and ICU mortality (AUROC = 0.76; 95% CI, 0.63–0.89).

**Table 3:** Predictors of 28-day mortality and ICU mortality in the sepsis cohort—univariable logistic regression models.

**Outcome variable**	**28-day mortality**			**ICU mortality**		
**Variable**	**Odds ratio**	**95% confidence interval**	***p***	**Odds ratio**	**95% confidence interval**	***p***
**Demographics and pre-medication**						
Age (per 5 years increase)	1.23	1.02–1.50	0.033	1.06	0.89–1.27	0.511
Body mass index (per 5 kg/m^2^ increase)	0.72	0.41–1.25	0.245	0.57	0.30–1.08	0.085
Female sex	2.71	0.87–8.42	0.085	1.65	0.53–5.17	0.390
Anti-diabetic therapy	0.75	0.20–2.75	0.665	0.87	0.22–3.37	0.836
Statin therapy	0.45	0.13–1.57	0.210	0.56	0.15–2.08	0.384
**Quantitative lipid parameters**						
HDL cholesterol (per 10 mg/l increase)	0.88	0.66–1.18	0.381	0.84	0.61–1.17	0.312
Triglycerides (per 10 mg/l increase)	0.98	0.93–1.04	0.501	0.99	0.93–1.05	0.715
Cholesterol (per 10 mg/l increase)	0.90	0.79–1.02	0.088	0.89	0.78–1.02	0.099
ApoA1 (per 10 mg/l increase)	0.92	0.80–1.07	0.294	0.87	0.73–1.03	0.099
**Qualitative lipid parameters**						
Arylesterase activity (AEA) (per 10 mM/min/ml serum increase)	0.76	0.61–0.94	0.010	0.71	0.56–0.90	0.004
Cholesterol efflux capacity (per 10% increase)	0.20	0.03–1.45	0.111	0.11	0.01–1.03	0.053
**Laboratory covariables**						
White blood count (per 1 g/L increase)	1.02	0.98–1.07	0.357	1.00	0.96–1.05	0.960
Hemoglobin (per 1 g/dl increase)	0.94	0.77–1.15	0.574	1.03	0.84–1.25	0.801
Platelets (per 100 g/L increase)	1.11	0.71–1.75	0.640	1.14	0.71–1.81	0.593
C-reactive protein (per 100 mg/l increase)	1.72	1.07–2.77	0.025	1.40	0.90–2.18	0.136
Serum bilirubin (per 1 mg/l increase)	0.89	0.74–1.08	0.245	0.94	0.80–1.11	0.484
Serum creatinine (per 1 mg/l increase)	1.01	0.85–1.19	0.905	1.04	0.88–1.23	0.653
Serum albumin (per 1 g/dl increase)	0.36	0.14–0.93	0.034	0.54	0.22–1.31	0.171
**Sepsis severity and outcomes**						
SOFA score (per 1 point increase)	1.13	0.97–1.31	0.113	1.36	1.12–1.65	0.002

*AEA, arylesterase activity; ApoA1, apolipoprotein A-I; HDL, high-density lipoprotein; SOFA, sequential organ failure assessment*.

**Table 4:** Two multivariable models for 28-day and ICU mortality of the sepsis cohort.

	**Odds ratio**	**95% confidence**	***p***
		**interval**	
**Multivariable model 1: 28-day mortality**			
Arylesterase activity (AEA)	0.76	0.59–0.98	0.032
(per 10 mM/min/ml serum increase)			
SOFA score (per 1 point increase)	1.11	0.91–1.35	0.292
Age (per 5 years increase)	1.27	0.99–1.64	0.064
C-reactive protein	1.71	0.99–2.94	0.053
(per 100 mg/l increase)			
**Multivariable model 2: ICU mortality**			
Arylesterase activity (AEA) (per 10 mM/min/ml serum increase)	0.74	0.57–0.96	0.026
SOFA score (per 1 point increase)	1.30	1.06–1.59	0.010

*For multivariable analysis, we always considered the SOFA score and, additionally, all variables with a univariable p of association with the outcome of ≤ 0.05. Albumin was omitted from multivariable model 1 due to strong collinearity with C-reactive protein*.

*AEA, arylesterase activity; SOFA, sequential organ failure assessment*.

## Discussion

Our study assessed quantitative and qualitative changes of lipid parameters in patients with sepsis and septic shock admitted to the ICU and investigated differences to ICU controls without sepsis. We were also able to show that acute organ dysfunctions in sepsis lead to altered lipid profiles with low levels of HDL-C. Early sepsis symptoms and signs are unspecific and unreliable. The only biomarker mentioned in the surviving sepsis campaign is procalcitonin (PCT) on the discontinuation of antibiotics (21, 22). A PCT-guided antibiotic initiation or escalation strategy leads to higher rates of organ dysfunction, and the role of PCT was therefore questioned (23, 24). Better biomarkers are urgently needed, and this gap may be filled with the assessment of lipid parameters in patients with sepsis and septic shock.

HDL can bind LPS of Gram-negative bacteria and can aid in the neutralization of LTA from Gram-positive bacteria (25, 26). Further beneficial effects of HDL include the activation of eNOS leading to reduced vascular tension and inhibition of endothelial cell apoptosis (8, 11, 12, 27–29). As seen in our study, septic dyslipidemia is a common feature and present in 85% of all patients with sepsis and septic shock admitted to the ICU, while dyslipidemia was less prevalent in non-sepsis ICU patients. Tanaka et al. (30) showed that sepsis patients in a surgical ICU had lower HDL-C levels than trauma patients. ICU care in general (exemplarily for stress with inflammation) did not cause lipid levels as low as seen in sepsis (31). We found similar results in our study, as levels of HDL-C were severely reduced in sepsis patients with acute organ dysfunction at median 14 mg/l compared to non-sepsis ICU patients who had median HDL levels of 39 mg/l. HDL-C as a biomarker can easily be obtained in the routine laboratory and may aid in the diagnosis of sepsis. Furthermore, we found a statistically significant positive correlation between HDL-C and albumin only in the sepsis cohort, but not in ICU patients without sepsis. Our data are therefore in line with previous studies that reported hypoalbuminemia as a common finding in sepsis (32, 33).

ApoA1 is the main stabilizing apolipoprotein of HDL (34). ApoA1 itself can deactivate bacterial endotoxins and mitigate the TNF-alpha production after LPS or LTA exposure (35, 36). ApoA1 knockout mice have a higher risk of death from sepsis (7). In addition, reduced ApoA1 at ICU admission was associated with increasing SIRS levels during the ICU stay (37). Similarly, in our sepsis cohort, we were able to show that the SOFA score as a marker for disease severity correlated significantly with ApoA1 levels, and ApoA1 levels were reduced in septic patients.

Lipoproteins such as HDL decrease quickly in the acute phase and were found to be a negative prognostic marker in some studies. Lekkou et al. (38) showed that severe sepsis survivors had significantly higher HDL-C levels than non-survivors, and all patients with HDL-C >25 mg/l survived. In contrast, in another study, only SAPS2 score and ApoA1 levels were predictive for 30-day mortality (39). Cirstea et al. (40) found that HDL-C <25.1 mg/l was a negative prognostic marker for 90-day mortality. In line with the majority of these publications, we were able to show that HDL-C levels correlated with the disease severity assessed by SOFA score. However, we did not find quantitative numbers of HDL-C to be a significant predictor of 28-day or ICU mortality. This can be partially explained by the fact that we only included patients admitted to the ICU, whereas in the study of Cirstea et al. (40), only 48% needed ICU care. Similar to a study by Ngaosuwan et al. (41), we detected no significant alteration of serum triglyceride levels in sepsis patients.

Furthermore, in our study, the acute phase during sepsis led to a markedly altered effectiveness and functionality of HDL particles with significantly decreased AEA and CEC. In general, paraoxonase 1 (PON1) is a main contributor to the antioxidative properties of HDL (42). It can hydrolyze oxidized phospholipids, acts as a lactonase, and can neutralize homoserine lactones (e.g., from pseudomonas bacteria) (43–45). In a small study including ten patients in- and outside the ICU, septic patients had reduced CEC, but the activity of PON1 was not significantly different (16). A recent report showed that activity of PON1 was a good marker for the development of multiorgan dysfunction syndrome (46). Novak et al. (47) showed a non-significant trend of lower AEA in non-survivors of sepsis. In this regard, our study adds several interesting findings to the current knowledge. Patients with sepsis had reduced CEC at 9.2%, but this result was not statistically significantly different compared to non-sepsis ICU patients showing a CEC of 9.9%. On the other hand, sepsis patients compared to ICU controls had decreased AEA of PON1 (67 compared to 111 mM/min/ml serum). Additionally, we were able to show that the SOFA score as a measurement of severity and organ dysfunction correlated with the AEA in the sepsis cohort. AEA was also predictive for 28-day and ICU mortality in univariable and multivariable analyses in sepsis patients.

In general, simply raising HDL-C levels may not be beneficial in sepsis. As shown in the ILLUMINATE trial, there was increased infection-related mortality in patients treated with the cholesterylester transfer protein (CETP) inhibitor torcetrapib, despite an increase in HDL-C (48). The primary effect of CETP inhibition is a reduced transfer rate of cholesterylester from HDL into triglyceride rich lipoproteins. This leads to an increased content of cholesterylester in HDL and the formation of larger HDL particles that are more slowly catabolized than normal. Importantly, effects of CETP inhibitors on total number of HDL particles are weak, consistent with their weaker effects on plasma concentrations of ApoA1 (49). In addition, carriers of some mutation show efficient CEC despite having low HDL (50, 51). Therefore, the functionality of HDL could be of greater importance than the absolute number, as the robust associations of our study suggest.

### Limitations

First, our study had a single-center design, which may limit external validity. Second, lipid profiles may be influenced by glycemic control, which was not recorded in the study. However, our study, patients were not allocated to either conservative or intensive glycemic control but received standard care. Third, septic patients may have presented on different time spots within the disease. This is unfortunately a common and limiting factor of many, if not all, sepsis trials. Fourth, a major limitation of our study is the sample size. The SOFA score was a significant predictor for ICU mortality but not for 28-day mortality in our cohort. An only minimally larger sample size would have narrowed the 95% CI of the SOFA score regarding 28-day mortality and therefore likely provided a statistically significant association. Thus, our results need to be confirmed in a larger cohort.

## Conclusion

In conclusion, HDL functionality is profoundly altered during sepsis, implicating not only quantitative but also functional immunomodulatory alterations of HDL particles. Therefore, compositional but most importantly functional lipoprotein parameters may support clinicians to diagnose sepsis and predict severity and outcome at an early stage. In particular, the functionality parameter arylesterase activity of the HDL associated paraoxonase, which can be easily measured in clinical laboratories, shows promising potential for severity prediction and stratification in patients suffering from sepsis and septic shock.

## Data Availability Stateent

The raw data supporting the conclusions of this article will be made available by the authors, without undue reservation.

## Ethics Statement

The studies involving human participants were reviewed and approved by Institutional Review Board (IRB) of the Medical University of Graz, Austria (30-258 ex 17/18). The patients/participants provided their written informed consent to participate in this study.

## Author Contributions

AR and PE recruited patients. MS, JS, MH, GM, and AR performed the measurements. AR, FP, and PE analyzed the data, performed statistical analyses, and wrote the manuscript. GH, HS, RE, and KE critically revised the manuscript for important intellectual content. All authors approved the final version of the manuscript and agreed to be accountable for all aspects related to accuracy and integrity of the work.

## Conflict of Interest

The authors declare that the research was conducted in the absence of any commercial or financial relationships that could be construed as a potential conflict of interest.

## Abbreviations

ApoA1: apolipoprotein A-I
ApoB: apolipoprotein B
AUROC: area under the receiver operating characteristic curve
AEA: arylesterase activity of HDL-associated paraoxonase
ABCA1: ATP-binding cassette transporter A1
CEC: cholesterol efflux capacity
CETP: cholesterylester transfer protein
LPS: lipopolysaccharides
LTA: lipoteichoic acid
PON1: paraoxonase 1
PCT: procalcitonin
SOFA: sequential organ failure assessment.
