# Supplementary material for: Arylesterase Activity of HDL Associated Paraoxonase as a Potential Prognostic Marker in Patients With Sepsis and Septic Shock—A Prospective Pilot Study
Source: Front Med (Lausanne). 2020 Oct 22;7:579677. doi: 10.3389/fmed.2020.579677 (PMC7642222; doi:10.3389/fmed.2020.579677)
Supplement: Supplementary file 1 [file Data_Sheet_1.docx]

**Supplementary table X1:** Detailed report of sensitivity and specificity for predicting 28-day mortality with arylesterase activity

------------------------------------------------------------------------------

Correctly

Cutpoint Sensitivity Specificity Classified LR+ LR-

------------------------------------------------------------------------------

( >= .5 ) 100.00% 0.00% 47.17% 1.0000

( >= 6.4 ) 96.00% 0.00% 45.28% 0.9600

( >= 9.1 ) 96.00% 3.57% 47.17% 0.9956 1.1200

( >= 19.4 ) 92.00% 3.57% 45.28% 0.9541 2.2400

( >= 20.6 ) 88.00% 3.57% 43.40% 0.9126 3.3600

( >= 23.5 ) 84.00% 3.57% 41.51% 0.8711 4.4800

( >= 29.6 ) 80.00% 3.57% 39.62% 0.8296 5.6000

( >= 35 ) 76.00% 3.57% 37.74% 0.7881 6.7200

( >= 35.3 ) 76.00% 7.14% 39.62% 0.8185 3.3600

( >= 35.7 ) 72.00% 7.14% 37.74% 0.7754 3.9200

( >= 38.1 ) 68.00% 7.14% 35.85% 0.7323 4.4800

( >= 38.5 ) 64.00% 7.14% 33.96% 0.6892 5.0400

( >= 38.6 ) 64.00% 10.71% 35.85% 0.7168 3.3600

( >= 43.1 ) 64.00% 14.29% 37.74% 0.7467 2.5200

( >= 49.2 ) 60.00% 14.29% 35.85% 0.7000 2.8000

( >= 50.1 ) 56.00% 14.29% 33.96% 0.6533 3.0800

( >= 54.7 ) 56.00% 17.86% 35.85% 0.6817 2.4640

( >= 55.3 ) 52.00% 17.86% 33.96% 0.6330 2.6880

( >= 56.4 ) 52.00% 21.43% 35.85% 0.6618 2.2400

( >= 58.1 ) 52.00% 25.00% 37.74% 0.6933 1.9200

( >= 58.8 ) 52.00% 28.57% 39.62% 0.7280 1.6800

( >= 61.4 ) 52.00% 32.14% 41.51% 0.7663 1.4933

( >= 61.9 ) 52.00% 35.71% 43.40% 0.8089 1.3440

( >= 62.2 ) 48.00% 35.71% 41.51% 0.7467 1.4560

( >= 62.3 ) 44.00% 35.71% 39.62% 0.6844 1.5680

( >= 64.59.. ) 40.00% 35.71% 37.74% 0.6222 1.6800

( >= 66.5 ) 40.00% 39.29% 39.62% 0.6588 1.5273

( >= 68.40.. ) 40.00% 42.86% 41.51% 0.7000 1.4000

( >= 69.2 ) 40.00% 46.43% 43.40% 0.7467 1.2923

( >= 70.2 ) 36.00% 46.43% 41.51% 0.6720 1.3785

( >= 70.90.. ) 32.00% 46.43% 39.62% 0.5973 1.4646

( >= 72.90.. ) 28.00% 46.43% 37.74% 0.5227 1.5508

( >= 75.2 ) 24.00% 46.43% 35.85% 0.4480 1.6369

( >= 77.3 ) 24.00% 50.00% 37.74% 0.4800 1.5200

( >= 80.7 ) 20.00% 50.00% 35.85% 0.4000 1.6000

( >= 83.90.. ) 16.00% 50.00% 33.96% 0.3200 1.6800

( >= 84.90.. ) 16.00% 53.57% 35.85% 0.3446 1.5680

( >= 86.59.. ) 12.00% 53.57% 33.96% 0.2585 1.6427

( >= 88 ) 12.00% 57.14% 35.85% 0.2800 1.5400

( >= 88.7 ) 8.00% 57.14% 33.96% 0.1867 1.6100

( >= 90.3 ) 8.00% 60.71% 35.85% 0.2036 1.5153

( >= 92.8 ) 4.00% 60.71% 33.96% 0.1018 1.5812

( >= 94.40.. ) 4.00% 64.29% 35.85% 0.1120 1.4933

( >= 95.09.. ) 4.00% 67.86% 37.74% 0.1244 1.4147

( >= 96.2 ) 4.00% 71.43% 39.62% 0.1400 1.3440

( >= 98.09.. ) 4.00% 75.00% 41.51% 0.1600 1.2800

( >= 102.8 ) 4.00% 78.57% 43.40% 0.1867 1.2218

( >= 104.1 ) 4.00% 82.14% 45.28% 0.2240 1.1687

( >= 113 ) 0.00% 82.14% 43.40% 0.0000 1.2174

( >= 114.1 ) 0.00% 85.71% 45.28% 0.0000 1.1667

( >= 118.8 ) 0.00% 89.29% 47.17% 0.0000 1.1200

( >= 123 ) 0.00% 92.86% 49.06% 0.0000 1.0769

( >= 162.3 ) 0.00% 96.43% 50.94% 0.0000 1.0370

( > 162.3 ) 0.00% 100.00% 52.83% 1.0000

------------------------------------------------------------------------------

**Supplementary table X2:** Detailed report of sensitivity and specificity for predicting ICU mortality with arylesterase activity

------------------------------------------------------------------------------

Correctly

Cutpoint Sensitivity Specificity Classified LR+ LR-

------------------------------------------------------------------------------

( >= .5 ) 100.00% 0.00% 35.85% 1.0000

( >= 6.4 ) 94.74% 0.00% 33.96% 0.9474

( >= 9.1 ) 94.74% 2.94% 35.85% 0.9761 1.7895

( >= 19.4 ) 89.47% 2.94% 33.96% 0.9219 3.5789

( >= 20.6 ) 84.21% 2.94% 32.08% 0.8676 5.3684

( >= 23.5 ) 84.21% 5.88% 33.96% 0.8947 2.6842

( >= 29.6 ) 78.95% 5.88% 32.08% 0.8388 3.5789

( >= 35 ) 73.68% 5.88% 30.19% 0.7829 4.4737

( >= 35.3 ) 73.68% 8.82% 32.08% 0.8081 2.9825

( >= 35.7 ) 68.42% 8.82% 30.19% 0.7504 3.5789

( >= 38.1 ) 63.16% 8.82% 28.30% 0.6927 4.1754

( >= 38.5 ) 57.89% 8.82% 26.42% 0.6350 4.7719

( >= 38.6 ) 57.89% 11.76% 28.30% 0.6561 3.5789

( >= 43.1 ) 57.89% 14.71% 30.19% 0.6788 2.8632

( >= 49.2 ) 52.63% 14.71% 28.30% 0.6171 3.2211

( >= 50.1 ) 47.37% 14.71% 26.42% 0.5554 3.5789

( >= 54.7 ) 47.37% 17.65% 28.30% 0.5752 2.9825

( >= 55.3 ) 42.11% 17.65% 26.42% 0.5113 3.2807

( >= 56.4 ) 42.11% 20.59% 28.30% 0.5302 2.8120

( >= 58.1 ) 42.11% 23.53% 30.19% 0.5506 2.4605

( >= 58.8 ) 42.11% 26.47% 32.08% 0.5726 2.1871

( >= 61.4 ) 42.11% 29.41% 33.96% 0.5965 1.9684

( >= 61.9 ) 42.11% 32.35% 35.85% 0.6224 1.7895

( >= 62.2 ) 36.84% 32.35% 33.96% 0.5446 1.9522

( >= 62.3 ) 36.84% 35.29% 35.85% 0.5694 1.7895

( >= 64.59.. ) 31.58% 35.29% 33.96% 0.4880 1.9386

( >= 66.5 ) 31.58% 38.24% 35.85% 0.5113 1.7895

( >= 68.40.. ) 31.58% 41.18% 37.74% 0.5368 1.6617

( >= 69.2 ) 31.58% 44.12% 39.62% 0.5651 1.5509

( >= 70.2 ) 26.32% 44.12% 37.74% 0.4709 1.6702

( >= 70.90.. ) 21.05% 44.12% 35.85% 0.3767 1.7895

( >= 72.90.. ) 21.05% 47.06% 37.74% 0.3977 1.6776

( >= 75.2 ) 15.79% 47.06% 35.85% 0.2982 1.7895

( >= 77.3 ) 15.79% 50.00% 37.74% 0.3158 1.6842

( >= 80.7 ) 10.53% 50.00% 35.85% 0.2105 1.7895

( >= 83.90.. ) 5.26% 50.00% 33.96% 0.1053 1.8947

( >= 84.90.. ) 5.26% 52.94% 35.85% 0.1118 1.7895

( >= 86.59.. ) 5.26% 55.88% 37.74% 0.1193 1.6953

( >= 88 ) 5.26% 58.82% 39.62% 0.1278 1.6105

( >= 88.7 ) 5.26% 61.76% 41.51% 0.1377 1.5338

( >= 90.3 ) 5.26% 64.71% 43.40% 0.1491 1.4641

( >= 92.8 ) 0.00% 64.71% 41.51% 0.0000 1.5455

( >= 94.40.. ) 0.00% 67.65% 43.40% 0.0000 1.4783

( >= 95.09.. ) 0.00% 70.59% 45.28% 0.0000 1.4167

( >= 96.2 ) 0.00% 73.53% 47.17% 0.0000 1.3600

( >= 98.09.. ) 0.00% 76.47% 49.06% 0.0000 1.3077

( >= 102.8 ) 0.00% 79.41% 50.94% 0.0000 1.2593

( >= 104.1 ) 0.00% 82.35% 52.83% 0.0000 1.2143

( >= 113 ) 0.00% 85.29% 54.72% 0.0000 1.1724

( >= 114.1 ) 0.00% 88.24% 56.60% 0.0000 1.1333

( >= 118.8 ) 0.00% 91.18% 58.49% 0.0000 1.0968

( >= 123 ) 0.00% 94.12% 60.38% 0.0000 1.0625

( >= 162.3 ) 0.00% 97.06% 62.26% 0.0000 1.0303

( > 162.3 ) 0.00% 100.00% 64.15% 1.0000

------------------------------------------------------------------------------
